# Gastric-Type Adenocarcinoma of the Uterine Cervix Associated with Poor Response to Definitive Radiotherapy

**DOI:** 10.3390/cancers15010170

**Published:** 2022-12-28

**Authors:** Airi Kuruma, Michiko Kodama, Yumiko Hori, Kazuaki Sato, Makoto Fujii, Fumiaki Isohashi, Ai Miyoshi, Seiji Mabuchi, Akira Setoguchi, Hiroko Shimura, Takeshi Goto, Aska Toda, Satoshi Nakagawa, Yasuto Kinose, Tsuyoshi Takiuchi, Eiji Kobayashi, Kae Hashimoto, Yutaka Ueda, Kenjiro Sawada, Eiichi Morii, Tadashi Kimura

**Affiliations:** 1 Department of Obstetrics and Gynecology, Osaka University Graduate School of Medicine, 2-2, Yamadaoka, Suita City 5650871, Osak, Japan; 2 Department of Central Laboratory and Surgical Pathology, National Hospital Organization Osaka National Hospital, 2-1-4, Hoenzaka, Chuo-Ku, Osaka City 5400006, Osaka, Japan; 3 Department of Pathology, Osaka University Graduate School of Medicine, 2-2, Yamadaoka, Suita City 5650871, Osaka, Japan; 4 Division of Health Science, Osaka University Graduate School of Medicine, 1-7, Yamadaoka, Suita City 5650871, Osaka, Japan; 5 Department of Radiation Oncology, Osaka University Graduate School of Medicine, 2-2, Yamadaoka, Suita City 5650871, Osaka, Japan; 6 Department of Gynecology, Osaka International Cancer Institute, 3-1-69, Otemae 5418567, Osaka, Japan; 7 Department of Obstetrics and Gynecology, Nippon Life Hospital, 2-1-54, Enokojima, Nishi-Ku, Osaka City 5500006, Osaka, Japan

**Keywords:** cervical cancer, gastric-type adenocarcinoma, definitive radiotherapy

## Abstract

**Simple Summary:**

Cervical cancer is the most common frequent gynecological malignancy. The incidence has decreased owing to screening programs and human papillomavirus vaccination; however, the incidence of adenocarcinoma has recently increased in some countries, especially in the young population. Adenocarcinoma treated with definitive radiotherapy, the recommended approach for treating locally advanced cervical cancer, has a lower response and survival rate than squamous cell carcinoma. Our study aimed to assess the response to definitive radiotherapy by histological subtype and to investigate prognostic factors of adenocarcinoma according to the uniform staging system and histological classification. We confirmed that 52 patients with adenocarcinoma responded significantly less to definitive radiotherapy and had shorter survival times than 275 patients with squamous cell carcinoma. In the adenocarcinoma population, univariate and multivariate analyses showed that gastric-type adenocarcinoma was an independent poor prognostic factor associated with response to definitive radiotherapy. The pathogenesis of gastric-type adenocarcinoma must be investigated to overcome its poor response to treatment and establish novel therapeutic strategies for it.

**Abstract:**

We aimed to evaluate the response to definitive radiotherapy (RT) for cervical cancer based on histological subtypes and investigate prognostic factors in adenocarcinoma (AC). Of the 396 patients treated with definitive RT between January, 2010 and July, 2020, 327 patients met the inclusion criteria, including 275 with squamous cell carcinoma (SCC) and 52 with AC restaged based on the 2018 International Federation of Gynecology and Obstetrics staging system. Patient characteristics, response to RT, and prognoses of SCC and AC were evaluated. The complete response (CR) rates were 92.4% and 53.8% for SCC and AC, respectively (*p* < 0.05). Three-year overall survival and progression-free survival (PFS) rates of SCC were significantly higher than those of AC (88.6% vs. 74.1%, *p* < 0.05 and 76.3% vs. 59.3%, *p* < 0.05, respectively). Among the AC population, univariate and multivariate analyses were performed to examine prognostic factors associated with non-complete response (CR). In the multivariate analysis, gastric-type adenocarcinoma (GAS) was associated with non-CR in AC (adjusted odds ratio, 12.2; 95% confidence interval 1.0–145.6; *p* < 0.05). The 3-year PFS rate in patients with GAS was significantly lower than that in patients with other histological types of AC (44.4% vs. 66.7%, *p* < 0.05). Definitive RT for cervical cancer was significantly less effective for AC than for SCC. GAS was the only independent prognostic factor associated with non-CR in AC.

## 1. Introduction

Cervical cancer is the fourth most common cancer among women worldwide, according to GLOBOCAN 2020. An estimated 604,000 women were diagnosed with cervical cancer and approximately 342,000 died in 2020 [1]. Globally, the human papillomavirus (HPV) vaccine and cancer screening programs have reduced the incidence and mortality of cervical cancer in countries categorized as having a high human development index [2,3]. However, the incidence of cervical cancer has increased in some European countries and Japan, especially in the young populations [4,5,6]. Changes in sexual lifestyle, the spread of HPV infection in younger women, and the consistently low rate of cervical cancer screening are all considered causes of this increased incidence [5]. Squamous cell carcinoma (SCC) has been the most common histological type of cervical cancer. While the incidence of adenocarcinoma (AC) was only approximately 5% during the 1950s and 1960s, it has recently increased to 25% [7]. This trend has also been reported in Japan, particularly in younger age groups [5], where the rate of AC was 18% in 2006–2010 versus 4% in 1976–1980, and the annual percentage change was 5.0% in the age group ≤ 39 years in 1976–2012 [5].

According to the National Comprehensive Cancer Network Clinical Practice Guidelines in Oncology [8] and the European Society for Medical Oncology guidelines [9], definitive radiotherapy (RT) is recommended for locally advanced cervical cancer regardless of the histological type. This is due to the lack of solid evidence regarding the prognostic differences between the treatment modalities of SCC and AC. While the histologic impact on the survival of locally advanced cervical cancer remains controversial, several pieces of evidence have been accumulated. AC is considered less radiosensitive because AC treated with definitive RT alone showed poorer survival than SCC; however, radiosensitizers improved the survival outcome in AC to a level similar to that in SCC [10]. Indeed, in locally advanced cervical cancer, AC and SCC had similar survival outcomes when concurrent chemoradiation (CCRT) was employed [10,11]; however, some reports have shown that AC treated with CCRT had a worse survival rate than SCC [12,13,14]. The mechanism of poor prognosis in AC remains unclear. Nevertheless, larger tumor size, older age, and incomplete response to RT are the prognostic factors for it [13].

HPV infection, the leading cause of cervical cancer, is known to be less frequent in AC than in SCC [15,16]. Most SCC cases are associated with HPV infection, while 85–90% of AC cases are HPV-associated [17,18]. HPV-independent cervical cancer is more aggressive and has a worse prognosis than HPV-associated cancer [17]. Therefore, the fifth edition of the 2020 World Health Organization (WHO) classification of cervical cancer categorizes epithelial tumors and their precursors according to their association with HPV infection [19]. The most common HPV-independent cervical cancer, gastric-type adenocarcinoma (GAS), is a subtype of endocervical mucinous adenocarcinoma that was added to the WHO classification in 2014 [20]. GAS, first described in 2007, is characterized by a distinctive morphology of (1) clear or pale eosinophilic cytoplasm, (2) voluminous cytoplasm, and (3) distinct cell borders [21]. GAS is immunochemically positive for HIK1083, MUC6, or both, estrogen and progesterone receptors are usually negative, p16 is negative or focally positive, and mutant p53 is positive in approximately 50% of the cases [22]. GAS accounts for 9.7–28.9% of all endocervical adenocarcinoma in Japan [23,24] but is rare in western countries, with a prevalence of 1.5% [25]. GAS is an aggressive treatment-resistant cancer. Therefore, its prognosis is worse than that of usual endocervical adenocarcinoma (UEA), even in early-stage cancers that can be treated with radical hysterectomy [26,27]. 

In this study, we reevaluated cases from the past 10 years based on the same updated staging and, for AC, pathologic classification to evaluate the response to definitive RT and survival outcome by histological type. We further examined the factors associated with its poor response and prognosis in AC.

## 2. Materials and Methods

### 2.1. Study Sample

Patients who underwent definitive RT for International Federation of Gynecology and Obstetrics (FIGO) 2018 stage IA2-IVA cervical cancer, including a downstaged case of FIGO 2008 IB1, at Osaka University Hospital between January, 2010 and July, 2020, were reviewed retrospectively (Table S1). This study was approved by the institutional review board of Osaka University, and informed consent was obtained as an opt-out on the website. 

### 2.2. Study Data

The patients’ demographic characteristics, including age, parity, body mass index, pretreatment laboratory data, hemoglobin, neutrophil–lymphocyte ratio (NLR), tumor size calculated using magnetic resonance imaging (MRI) or transvaginal ultrasonography, histopathology, tumor staging reclassified according to FIGO 2018, lymph node metastasis, radiosensitizers, treatment outcome, and prognosis, were extracted from electronic medical records. Two investigators performed tumor restaging based on imaging examinations. The prognosis was confirmed on 30 November 2021. The exclusion criteria were as follows: histological type except for SCC, AC, and adenosquamous carcinoma (ASC); incomplete RT due to its adverse effects; and unconfirmed therapeutic effect of RT due to loss of follow-up. ASC cases were included in the AC group for the analysis.

The response to RT was assessed using MRI and computed tomography (CT) within approximately 3 months after RT completion. For cases of suspected persistent disease, MRI, histological examination, and positron emission tomography (PET)-CT were performed within 2 months to decide whether to perform salvage surgery or initiate chemotherapy. Complete response (CR) was defined as the absence of tumors on MRI or PET-CT, or when the persistent disease was not diagnosed based on histological examination. Partial response (PR), stable disease, or progressive disease (PD) was defined according to the response evaluation criteria in solid tumors.

As previously reported, patients underwent regular follow-ups by both gynecological and radiation oncologists [28].

Local recurrence was defined as recurrence in the cervix or vagina; regional recurrence was defined as recurrence in the pelvis, excluding the cervix and vagina; and distant recurrence was defined as recurrence outside the pelvis, such as the liver or lung. Distant recurrence included paraaortic lymph node recurrence when the paraaortic lymph nodes were not within the irradiated field during the primary treatment. In analyzing the response to definitive RT, the cut-off value for tumor size was 5 cm, based on the size at which our SCC data could predict CR and non-CR. A high NLR was determined more than 2.5 based on the results of previous studies [29].

### 2.3. Definitive RT

Definitive RT comprised external beam radiotherapy (EBRT) and high-dose-rate (HDR) brachytherapy with platinum-based chemotherapy, as described previously [30,31]. Patients aged 75 years or older, with renal failure, or with an allergy to platinum agents, were treated without chemotherapy. EBRT was delivered via CT-based treatment planning at a dose of 2 Gy per fraction. The clinical target volume (CTV) included the gross tumor volume, cervix, parametria, uterus, upper part of the vagina, and regional lymph nodes (common, presacral, and internal and external iliac). Paraaortic lymph node RT was performed in cases with paraaortic nodal involvement. The dose of whole-pelvic RT (WPRT) before central shielding (CS) depended on the initial tumor size; patients with tumors < 4 cm and ≥4 cm in diameter received 30 Gy and 40 Gy, respectively, as the minimum dose. The initial 30–40 Gy was delivered using WPRT with a four-field box, and pelvic irradiation was then delivered with a 4 cm-wide CS. The total pelvic sidewall dose was 50 Gy. If it was unlikely that the tumor volume would be irradiated by intracavitary brachytherapy (ICBT) after 40 Gy, an additional whole-pelvic dose of 10 Gy without CS was delivered. An additional EBRT boost of 6–10 Gy was administered to patients with pelvic lymph nodes > 25 mm in diameter. After adequate tumor regression, HDR-ICBT was performed once a week during and after EBRT with CS. Four, three, and two fractions of ICBT were administered to the patients who received WPRT at 30, 40, and 50 Gy, respectively. Template-based interstitial brachytherapy was performed in patients with large or complex tumors. ICBT was administered via CT-based planning. A planning CT scan was performed before the delivery of each fraction. The high-risk CTV (HR-CTV) and organs at risk (OARs) were contoured on planning CT. The dwell times were manually modified using graphical optimization to maximize the coverage of the HR-CTV while reducing the dose to the OARs to meet our dose constraints: HR-CTV D90 > 6.0 Gy, rectum D2cc < 7.0 Gy, and bladder D2cc < 7.0 Gy.

### 2.4. Histological Reevaluation

AC cases, including ASC, were reevaluated by pathologists specializing in gynecological oncology and diagnosed according to the WHO 2020 classification of uterine cervical cancer (Table S2). Histological diagnosis was warranted based on hematoxylin and eosin (H&E) staining morphology and immunohistological findings of p16, p53, MUC6, p40 estrogen receptor, and Napsin A. Positive p16 was considered HPV-associated cancer. Eleven cases were excluded from the risk factor analysis for AC because of the lack of histological specimens.

### 2.5. Statistical Analysis

Categorical variables were analyzed using the Fisher’s exact probability test or Pearson’s chi-square test, and continuous variables were analyzed using the Mann–Whitney U test. Statistical significance was set at *p* < 0.05. Univariate logistic regression analysis was used to evaluate potential predictive factors associated with RT. Multivariate logistic regression analysis was conducted by selecting variables with *p* < 0.05 detected in univariate analysis or factors considered clinically significant. Overall survival (OS) and progression-free survival (PFS) were analyzed using the Kaplan–Meier method, and the significance of each survival difference was determined using log-rank test. All statistical analyses were performed using JMP®︎ Pro version 16.2.0 (SAS Institute Inc., Cary, NC, USA).

## 3. Results

**Figure 1:**
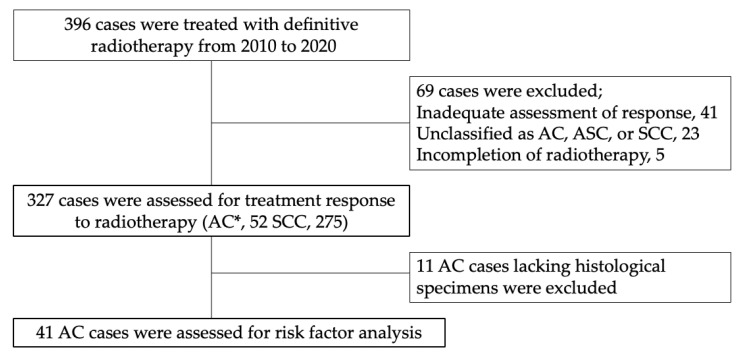
Flowchart of the analysis. AC* included two cases of ASC. Abbreviations: AC, adenocarcinoma; ASC, adenosquamous cell carcinoma; SCC, squamous cell carcinoma; CR, complete response.

A total of 396 patients were treated with definitive RT from 2010 to 2020. According to the inclusion and exclusion criteria, the remaining 327 patients, including 52 with AC (15.9%) and 275 with SCC (84.1%), were eligible for the analysis of their response to RT (Figure 1). The 69 patients excluded were as follows: 41 patients whose responses were not assessed due to lack of follow-up; 23 patients who were not classified as AC, ASC, or SCC; and 5 patients who did not complete RT due to side-effects. The 41 of 52 AC cases, excluding 11 cases lacking histological specimens, were further assessed for risk factors associated with response to RT and survival in AC.

The median follow-up period was 52.2 months (3.3–144.7), FIGO 2018 stage IIB and above was present in 243 patients (74.3%), lymph node metastasis was present in 140 patients (42.8%), and the median pretreatment tumor size was 4.3 cm (0.5–8.8) (Table 1). Patient characteristics, including pretreatment laboratory data, tumor stage and size, rate of lymph node metastasis, RT alone or CCRT, and rate of RT completion within 56 days, were not significantly different between AC and SCC. Of the 52 patients with AC, 28 (53.8%) and 12 (23.1%) achieved CR and PR, respectively. Of the 275 patients with SCC, 254 (92.4%) and 12 (4.4%) achieved CR and PR, respectively. AC responded significantly worse to RT compared with SCC (*p* < 0.05). Of the non-CR cases (24 AC cases and 21 SCC cases) after RT, 19 patients with AC (79.2% of non-CR cases) and 11 patients with SCC (52.4%) underwent salvage surgery. The remaining patients with non-CR received chemotherapy, RT for newly developed disease, or best supportive care. The recurrence rates of AC and SCC were not significantly different after CR was achieved (AC vs. SCC, 17.9% vs. 20.5%, *p* = 1.00). On the other hand, the recurrence rate, in all patients except cases defined as PD, was higher in AC (AC vs. SCC, 39.6% vs. 21.1%, *p* < 0.05). The sites of all first recurrences were 15.8% and 25.9% for local recurrence, 15.8% and 12.1% for regional recurrence, and 68.4% and 60.3% for distant recurrence in AC and SCC.

Survival analysis revealed that AC had a significantly worse prognosis than SCC. Three-year OS rates were 74.1% (95% confidence interval [CI]: 59.7–84.7) for AC and 88.6% (95% CI: 84.1–92.0) for SCC (*p* < 0.05). Three-year PFS rate was 59.3% (95% CI: 45.2–72.1) in AC and 76.3% (95% CI: 70.7–81.0) in SCC (*p* < 0.05) (Figure S1A,B). Focusing on the stage, 3-year OS and PFS were similar for AC and SCC in stage IA–IIA (94.1% vs. 96.6%, *p* = 0.51; and 88.9% vs. 90.6%, *p* = 0.40, respectively); however, they were significantly different between the two groups in stage IIB–IVA (61.7% vs. 86.0%, *p* < 0.05; and 41.8% vs. 71.6%, *p* < 0.05, respectively) (Figure S1C–F).

**Figure 2:**
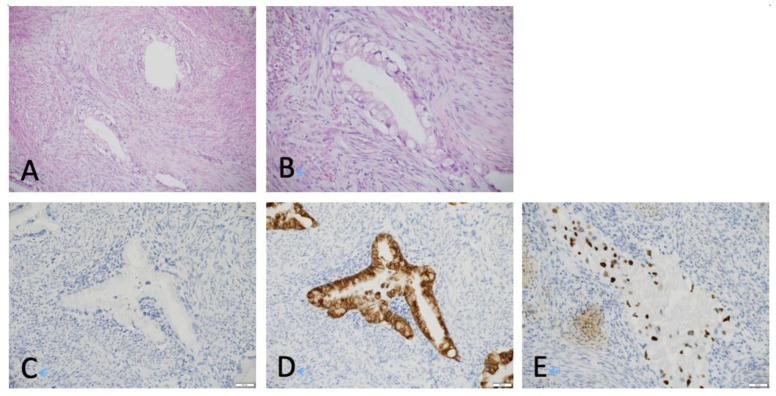
Representative pathological images of gastric-type adenocarcinoma of the uterine cervix. Tumors were stained with hematoxylin and eosin ((**A**), ×100, and (**B**), ×200), p16 ((**C**), ×200), MUC6 ((**D**), ×200), and p53 ((**E**), ×200). Scale bar showed 50 μm (**C**–**E**).

To elucidate the factors responsible for the poor response to RT, we further analyzed 41 of the 52 AC cases with histological reclassification, excluding 11 cases lacking histological specimens (Figure 1). The 41 AC cases were divided into CR and non-CR groups based on the evaluation of the response to definitive RT. There were no differences in the patient characteristics between the CR and non-CR groups (Table 2). The histopathological subclassification of patients with AC was 20 usual type, 9 GAS, 4 endometrioid, 3 poorly differentiated, 2 clear cell, and 2 ASC (Figure 2 and Figure S2). The remaining one case could not be reclassified because of the severe tissue degeneration. The proportion of patients with GAS, FIGO stage IIB and above, HPV-independent tumors, and high NLR was significantly higher in the non-CR group, whereas pretreatment tumor size was not.

Next, to identify the prognostic factors associated with non-CR in AC cases, univariate and multivariate analyses were performed after adjusting for confounding factors (Table 3). FIGO 2018 stage IIB and above, GAS, and high NLR were selected as significant prognostic factors by univariate logistic regression analysis, and tumor size, which was supposed to be a clinical prognostic predictive value, was set for the multivariate regression model. In the multivariate logistic regression analysis, GAS was independently associated with non-CR in AC (adjusted odds ratio, 12.2; 95% CI 1.0–145.6, *p* < 0.05).

Within the AC population, the 3-year OS rate in GAS tended to be lower than that in other histological types (50.0%, 95% CI: 20.0–80.0 vs. 78.4%, 95% CI: 59.4–90.0, *p* = 0.18), and the PFS rate was significantly lower in GAS (44.4%, 95% CI: 17.7–74.9 vs. 66.7%, 95% CI: 48.3–81.1, *p* < 0.05) (Figure S3 and Table S3). Patients with FIGO stage IIB and above, lymph node metastasis, and non-CR showed significantly lower 3-year OS rates than patients with FIGO stage <IIB, no lymph node metastasis, and CR, respectively (58.5% vs. 92.9%, *p* < 0.05; 58.7% vs. 78.9%, *p* < 0.05; 48.8% vs. 94.4%, *p* < 0.05, respectively).

Regarding the survival rate of patients with GAS, the CR rate was 11.1% (1/9). All patients with non-CR underwent salvage surgery, leading to complete surgery in seven cases, except for one incomplete case. After a median follow-up period of 22.2 months, seven patients who underwent surgery subsequently developed local recurrence in one case and distant recurrence in five cases. Eventually, five of the nine patients with GAS (55.6%) died, four of whom died within 2 years of the primary RT.

## 4. Discussion

The main findings of this study were that cervical adenocarcinoma responded poorly to definitive RT and that within the AC population, GAS negatively impacted treatment response and survival. Irrespective of the similar background, AC had a significantly worse CR rate to definitive RT compared with the CR rate of SCC (53.8% vs. 92.4%, respectively). AC had significantly lower 3-year OS and PFS rates than SCC, especially in stage IIB and above populations.

Several studies have reported the efficacy of definitive RT for locally advanced cervical cancer according to histologic type. A retrospective analysis of 182 patients with AC with stage IB2–IVA treated with definitive RT using cisplatin as a radiosensitizer had a similar OS to the OS of 1,489 patients with SCC; however, worse OS was observed with RT alone, according to combined GOG randomized trials of CCRT [10]. In this GOG study, AC tended to be more often stage IB2 and less frequently stage IIIB than SCC, and the tumor size was significantly smaller than that in SCC. Another study was conducted at a single institute and evaluated AC and SCC at stage IIB−IVA with 1:2 matching by stage, RT alone or CCRT, and tumor size [11]. The CR rate was significantly worse in 141 patients with AC than the CR rate in 282 patients with SCC (86.5% and 94.7%, respectively). However, the 5-year OS rates were similar in AC and SCC (59.9% and 61.7%, respectively). Even with the introduction of CCRT for locally advanced cervical cancer, AC remains a negative independent prognostic factor for survival (hazard ratio [HR], 1.40; 95% CI, 1.30–1.50) and is associated with poorer OS [12]. A recent systematic review and meta-analysis of eight studies analyzed 13,859 patients and showed that disease-free survival (HR, 1.51; 95% CI, 1.28–1.79) and OS (HR, 1.41; 95% CI, 1.26–1.57) of AC treated with definitive RT were lower than those of SCC [32]. Together with these findings, it can be surmised that locally advanced cervical adenocarcinoma responds less to RT than SCC, resulting in lower survival outcomes.

One reason for the low response rate in this study to definitive RT for cervical adenocarcinoma may be the large prevalence of GAS, which has been identified as resistant to current anticancer therapies. For early-stage GAS, some evidence showed resistance to chemotherapy or RT [26,27]; however, to our knowledge, the treatment outcome and survival of GAS treated with definitive RT remains unclear. In this study, GAS resistance to RT appeared to be strong enough to outweigh the effects of advanced stage and NLR, which are considered apparent risk factors associated with poor response to RT. GAS is more aggressive than UEA and is associated with several unfavorable prognostic factors, including bulky mass, deep stromal invasion, lymphovascular invasion, parametrial invasion, ovarian metastasis, positive ascitic cytology, pelvic lymph node metastasis, and advanced-stage cancer [33]. A phase II study was conducted to investigate the efficacy of neoadjuvant docetaxel/carboplatin combination chemotherapy followed by radical hysterectomy in 61 patients with FIGO 2009 stage IB2, IIA2, or IIB non-SCC. The response rate of GAS to chemotherapy was significantly lower than that of UEA (46.2% [6/13] vs. 85.0% [17/20], *p* = 0.048). The 5-year PFS rates and OS rates were lower in patients with GAS (38.5% vs. 75.0%, *p* = 0.011, and 36.9% vs. 90.0%, *p* < 0.001, respectively) [27]. In a retrospective study of 393 patients with FIGO 1988 stage IA–IIB endocervical cancer, including 95 patients with GAS who underwent radical hysterectomy without neoadjuvant chemotherapy, GAS recurred significantly more frequently than UEA (40.0% vs. 14.6%, *p* < 0.001). Regarding recurrence, the response to chemotherapy was not significantly different between GAS and UEA; however, the response to RT was significantly poorer in GAS than in UEA (50.0% vs. 81.7%, *p* < 0.0001) [26], supporting our findings that definitive RT for GAS showed a poor response rate.

It is unclear why different histologic types of cervical adenocarcinoma respond differently to RT, and it may be beneficial to explore the molecular mechanisms that may help establish new therapeutics for AC, including GAS. The development and affordability of new-generation sequencing methods have contributed to the elucidation of the carcinogenic mechanisms of cancers. The Cancer Genome Atlas (TCGA) project has revealed the genomic profiles of various cancers, including cervical cancer [34]. Focusing on the 31 patients with AC included in this study, *ERBB2* (28% of AC) and *ERBB3* (16%) alterations tended to co-occur vigorously and more frequently than in patients with SCC. However, the most common histological type of AC was endocervical adenocarcinoma, and the number of GAS cases included in this study is uncertain [34]. Recently, sporadic reports have emerged regarding the patterns of genetic variation in GAS. Target sequencing of 68 patients with GAS revealed somatic mutations in *TP53* (41%), *CDKN2A* (18%), *KRAS* (18%), and *STK11* (10%), and potentially targetable mutations in *ERBB3* (10%), *ERBB2* (8%), and *BRAF* (4%). Germline mutations in *STK11* are known to cause Peutz–Jeghers syndrome and are linked to up to 10% of GAS cases [35]. Cell cycle-related genes, including *TP53* and *CDKN2A*, are more affected in GAS than in UEA [36]. Epithelial-mesenchymal transition (EMT)-related genes were frequently mutated in GAS compared to SCC and endocervical adenocarcinoma in the TCGA dataset [37]. Taken together, these facts suggest that *TP53* is the most frequently mutated gene in GAS, and that *ERBB2/3* and *BRAF* are potential targets for molecularly-targeted drugs. EMT and cell cycle, well-known mechanisms of cancer progression and resistance to therapy, may be involved in the progression of GAS. 

The strength of this study is that it included advanced cervical cancer cases who underwent definitive RT at a single institution with a uniform treatment regimen and method. Additionally, all histopathology and staging were reviewed based on uniform criteria. New insights were provided in examining the treatment response and survival without surgery in patients with GAS treated with definitive RT.

However, this study has three main limitations. First, it was conducted at a single institution and the sample size was insignificant. The statistical power to determine the difference between SCC and AC, and between non-CR and CR in AC cases, was insufficient due to the sample size. Therefore, the differences shown in this study might be clinically informative but not statistically significant. Further large-scale studies are necessary to obtain a truly statistically significant difference. Second, due to the retrospective nature of the study, potential confounding biases might not have been excluded, such as selection bias introduced by physicians when determining the treatment modality. Cases were excluded from the analysis owing to a lack of clinical outcomes. Third, as this study covered an extended period, changes in the pretreatment work-up, diagnostic procedures, improvements in RT procedures, and surgical techniques for recurrent disease might have affected patient survival.

This study suggests that locally advanced cervical adenocarcinoma, especially GAS, responds less to definitive RT than SCC and has poor survival outcomes. Current and future advances in genomics, proteomics, and metabolomics will help elucidate the molecular mechanisms of treatment-resistant tumors. New treatment strategies, including molecular targeted therapies, are expected to be developed.

## 5. Conclusions

We demonstrated that the response to definitive RT was poorer in patients with AC than in those with SCC. In the multivariate analysis, GAS was identified as an independent variable associated with non-CR to definitive RT in patients with AC. Further investigation of the effect of definitive RT on GAS should be performed in a large-scale study.

## Figures and Tables

**Table 1:** Patients’ characteristics and treatment outcomes (AC vs. SCC).

Variables	Total (*n* = 327)		AC (*n* = 52)		SCC (*n* = 275)		*p*-Value
Follow-up time (months)	52.2	(3.3–144.7)	47.3	(3.5–100.3)	53	(3.3–144.7)	0.25
Patients’ characteristics							
Age (year)	59	(26–93)	57	(30–78)	61	(26–93)	0.12
Age ≧ 60	163	(49.8)	21	(40.4)	142	(51.6)	0.14
Non-parous *	74	(22.6)	12	(23.1)	62	(22.9)	0.98
BMI (kg/m^2^)	21.4	(13.3–40.6)	21.7	(16.6–40.6)	21.2	(13.3–40.1)	0.28
FIGO stage							
I	55	(16.8)	13	(25.0)	42	(15.3)	0.24
II	100	(30.6)	16	(30.8)	84	(30.5)	
III	163	(49.8)	23	(44.2)	140	(50.9)	
IV	9	(2.8)	0	(0.0)	9	(3.3)	
FIGO 2018 stage ≧ IIB	243	(74.3)	34	(65.4)	209	(76.0)	0.11
Lymph node metastasis	140	(42.8)	20	(38.5)	120	(43.6)	0.49
Tumor size (cm)	4.3	(0.5–8.8)	4.0	(1.0–8.8)	4.3	(0.5–8.2)	0.58
Tumor size ≧ 5 cm **	102	(32.4)	14	(28.6)	88	(33.1)	0.54
Pretreatment laboratory data							
Hemoglobin level (g/dl)	12.5	(5.8–15.8)	12.5	(8.0–15.8)	12.5	(5.8–15.8)	0.84
NLR	2.6	(0.6–24.6)	2.6	(0.9–7.6)	2.6	(0.6–24.6)	0.66
NLR ≧ 2.5	157	53.0	27	(56.3)	130	(43.9)	0.63
Type of treatment							
RT alone	74	(22.6)	11	(21.2)	63	(22.9)	0.78
CCRT	253	(77.4)	41	(78.8)	212	(77.1)	
RT completion within 56 days	317	(96.9)	49	(94.2)	268	(97.5)	0.20
Treatment outcome							
CR	282	(86.2)	28	(53.8)	254	(92.4)	<0.05
PR	24	(7.3)	12	(23.1)	12	(4.4)	
SD	11	(3.4)	8	(15.4)	3	(1.1)	
PD	10	(3.1)	4	(7.7)	6	(2.2)	
Recurrence after CR	57/282	(20.2)	5/28	(17.9)	52/254	(20.5)	1.00

Median (range) or n (%) are shown. * SCC had four missing data cases. ** AC had three cases and SCC had nine cases of missing data. Abbreviations: AC, adenocarcinoma; SCC, squamous cell carcinoma; BMI, body mass index; FIGO, International Federation of Gynecology and Obstetrics; NLR, neutrophil-lymphocyte ratio; RT, radiotherapy; CCRT, concurrent chemoradiotherapy; CR, complete response; PR, partial response; SD, stable disease; PD, progressive disease.

**Table 2:** The comparison between CR cases and non-CR cases in patients with AC.

Variables	AC (*n* = 41)				
	CR (*n* = 20)		Non-CR (*n* = 21)		*p*-Value
Patients’ characteristics					
Age (year)	55	(30–70)	58	(38–78)	1.00
Age ≧ 60	9	(45.0)	7	(33.3)	0.44
Non-parous	7	(35.0)	3	(14.3)	0.16
BMI (kg/m^2^)	21.0	(16.6–37.0)	22.0	(17.2–29.6)	0.38
Histopathology					
Gastric type	1	(5.0)	8	(38.1)	<0.05
Other type	19	(95.0)	13	(61.9)	
HPV status					
Associated	14	(70.0)	5	(23.8)	<0.05
Independent	4	(20.0)	11	(52.4)	
Undetermined	2	(10.0)	5	(23.8)	
FIGO stage ≧ IIB	8	(40.0)	18	(85.7)	<0.05
Lymph node metastasis	4	(20.0)	9	(42.9)	0.18
Tumor size (cm)	3.8	(1.0–6.6)	4.3	(1.7–8.8)	0.08
Tumor size ≧ 5.0 cm *	4	(23.5)	9	(42.9)	0.30
Pretreatment laboratory data					
Hemoglobin level (g/dl)	13.0	(8.5–15.8)	12.3	(8.0–14.2)	0.09
NLR	2.2	(1.5–4.6)	3.5	(0.9–7.6)	<0.05
NLR ≧ 2.5	8	(40.0)	16	(76.2)	<0.05
Treatment					
CCRT	14	(70.0)	18	(85.7)	0.28
RT alone	6	(30.0)	3	(14.3)	

Median (range) or n (%) are shown. * Three cases of missing data in the CR group. Abbreviations: CR, complete response; BMI, body mass index; HPV, human papillomavirus; FIGO, International Federation of Gynecology and Obstetrics; NLR, neutrophil-lymphocyte ratio; CCRT, concurrent chemoradiotherapy; RT, radiotherapy.

**Table 3:** Univariate and multivariate analyses for the factors related to non-CR in AC.

	*n*	Univariate Analysis		Multivariate Analysis *	
Variables	Non-CR/ Total (%)	cOR (95% CI)	*p*-Value	aOR (95% CI)	*p*-Value
Age			0.45		
<60	14/25 (56)	1			
≧60	7/16 (43.8)	0.6 (0.2–2.2)			
Parous			0.13		
nonparous	3/10 (30)	1			
multiparous	18/31 (58.1)	3.2 (0.7–14.9)			
Histology			<0.05		<0.05
Other type	13/32 (40.6)	1		1	
Gastric type	8/9 (88.9)	11.7 (1.3–105.0)		12.2 (1.0–145.6)	
FIGO stage			<0.05		0.10
<IIB	3/15 (20.0)	1		1	
≧IIB	18/26 (69.2)	9 (2.0–40.9)		4.8 (0.7–32.2)	
Lymph node metastasis			0.12		
No	12/28 (42.9)	1			
Yes	9/13 (69.2)	3.0 (0.7–12.1)			
Tumor size **			0.22		0.48
<5 cm	12/25 (48.0)	1		1	
≧5 cm	9/13 (69.2)	2.4 (0.6–10.0)		1.9 (0.3–10.3)	
NLR			<0.05		0.30
<2.5	5/17 (29.4)	1		1	
≧2.5	16/24 (66.7)	4.8 (1.3–18.4)		2.4 (0.5–12.2)	
Treatment			0.23		
RT alone	3/9 (33.3)	1			
CCRT	18/32 (56.3)	2.6 (0.5–12.1)			

* Adjusted for FIGO stage, histology, tumor size, and NLR. ** Three patients had missing data. Abbreviations: CR, complete response; AC, adenocarcinoma; cOR, crude odds ratio; aOR, adjusted odds ratio; CI, confidence interval; FIGO, International Federation of Gynecology and Obstetrics; NLR, neutrophil-lymphocyte ratio; RT, radiotherapy; CCRT, concurrent chemoradiotherapy.

## Data Availability

The data presented in this study are available upon reasonable request from the corresponding author.

## Supplementary Materials

The following supporting information can be downloaded at: https://www.mdpi.com/article/10.3390/cancers15010170/s1, Table S1: Details of International Federation of Gynecology and Obstetrics (FIGO) stage classification; Table S2: Detailed histopathology of adenocarcinoma (AC); Table S3: Three-year survival rates of patients with AC; Figure S1: Kaplan–Meier curves of overall survival (OS) and progression-free survival (PFS) by AC and squamous cell carcinoma (SCC). Figure S2: Representative pathological images of several types of cervical AC, including adenocarcinoma, usual type and clear-cell type, endometrioid adenocarcinoma, poorly differentiated adenocarcinoma, and adenosquamous cell carcinoma were shown. Figure S3: Kaplan–Meier curves of OS and PFS by gastric-type adenocarcinoma and other types of adenocarcinoma.
