# Supplementary material for: Gastric-Type Adenocarcinoma of the Uterine Cervix Associated with Poor Response to Definitive Radiotherapy
Source: Cancers (Basel). 2022 Dec 28;15(1):170. doi: 10.3390/cancers15010170 (PMC9818146; doi:10.3390/cancers15010170)
Supplement: Supplementary file 1 [file cancers-15-00170-s001.zip › cancers-2084486-supplementary.pdf]

Supplementary Table S1. The details of FIGO stage classified by AC and SCC

	Total (n=327)		AC (n=52)		SCC (n=275)	
	n (%)		n (%)		n (%)	
FIGO Stage						
IA2	1	(0.3)	0	(0.0)	1	(0.4)
IB1	22	(6.7)	7	(13.5)	15	(5.5)
IB2	22	(6.7)	2	(3.8)	20	(7.3)
IB3	10	(3.1)	4	(7.7)	6	(2.2)
IIA1	20	(6.1)	4	(7.7)	16	(5.8)
IIA2	10	(3.1)	2	(3.8)	8	(2.9)
IIB	70	(21.4)	10	(19.2)	60	(21.8)
IIIA	10	(3.1)	0	(0.0)	10	(3.6)
IIIB	20	(6.1)	4	(7.7)	16	(5.8)
IIIC1	98	(30.0)	13	(25.0)	85	(30.9)
IIIC2	35	(10.7)	6	(11.5)	29	(10.5)
IVA	9	(2.8)	0	(0.0)	9	(3.3)

Abbreviations: FIGO, International Federation of Gynecology and Obstetrics; AC, adenocarcinoma; SCC, squamous cell carcinoma.

Supplementary Table S2. The detailed histopathology of AC

	AC（n=41)			
	**CR (n=20)**		**non-CR (n=21)**	
	n (%)		n (%)	
Adenocarcinoma, usual type	11	(52.4)	9	(42.9)
Adenocarcinoma, gastric type	1	(4.8)	8	(38.1)
Adenocarcinoma, clear cell type	1	(4.8)	1	(4.8)
Endometrioid adenocarcinoma	2	(9.5)	2	(9.5)
Poorly differentiated adenocarcinoma	3	(14.3)	0	(0.0)
Adenosquamous carcinoma	2	(9.5)	0	(0.0)
Unclassifiable (degeneration)	0	(0.0)	1	(4.8)

Abbreviations: AC, adenocarcinoma; CR, complete response

Supplementary Table S3. The 3-year survival rate of patients with AC

	n	3-year survival rate (95%CI)	P-value
Age (year)			
≧ 60	16	59.5 (34.1–80.7)	0.06
< 60	25	81.1 (59.1–92.7)	
Histopathology			
Gastric type	9	50.0 (20.0–80.0)	0.18
Other type	32	78.4 (59.4–90.0)	
FIGO 2018 stage			
≧ IIB	26	58.5 (37.4–76.9)	<0.05
< IIB	15	92.9 (63.0–99.0)	
Lymph node status			
Metastasis	13	58.7 (31.1–81.8)	<0.05
No metastasis	28	78.9 (58.1–90.9)	
Tumor size (cm)*			
≧ 5cm	13	63.6 (33.9–85.7)	0.54
< 5cm	25	73.2 (51.5–87.5)	
NLR			
＜ 2.5	24	64.0 (41.2–81.8)	0.28
≧ 2.5	17	81.9 (56.4–94.1)	
Response to definitive radiotherapy			
CR	20	94.4 (69.3–99.2)	<0.05
non-CR	21	48.8 (27.0–71.0)	<0.05

*Three patients had missing data.

Abbreviations: AC, adenocarcinoma; CI, confidence interval; FIGO, International Federation of Gynecology and Obstetrics; NLR, neutrophil-to-lymphocyte ratio; CR, complete response; PR, partial response; SD, stable disease; PD, progressive disease

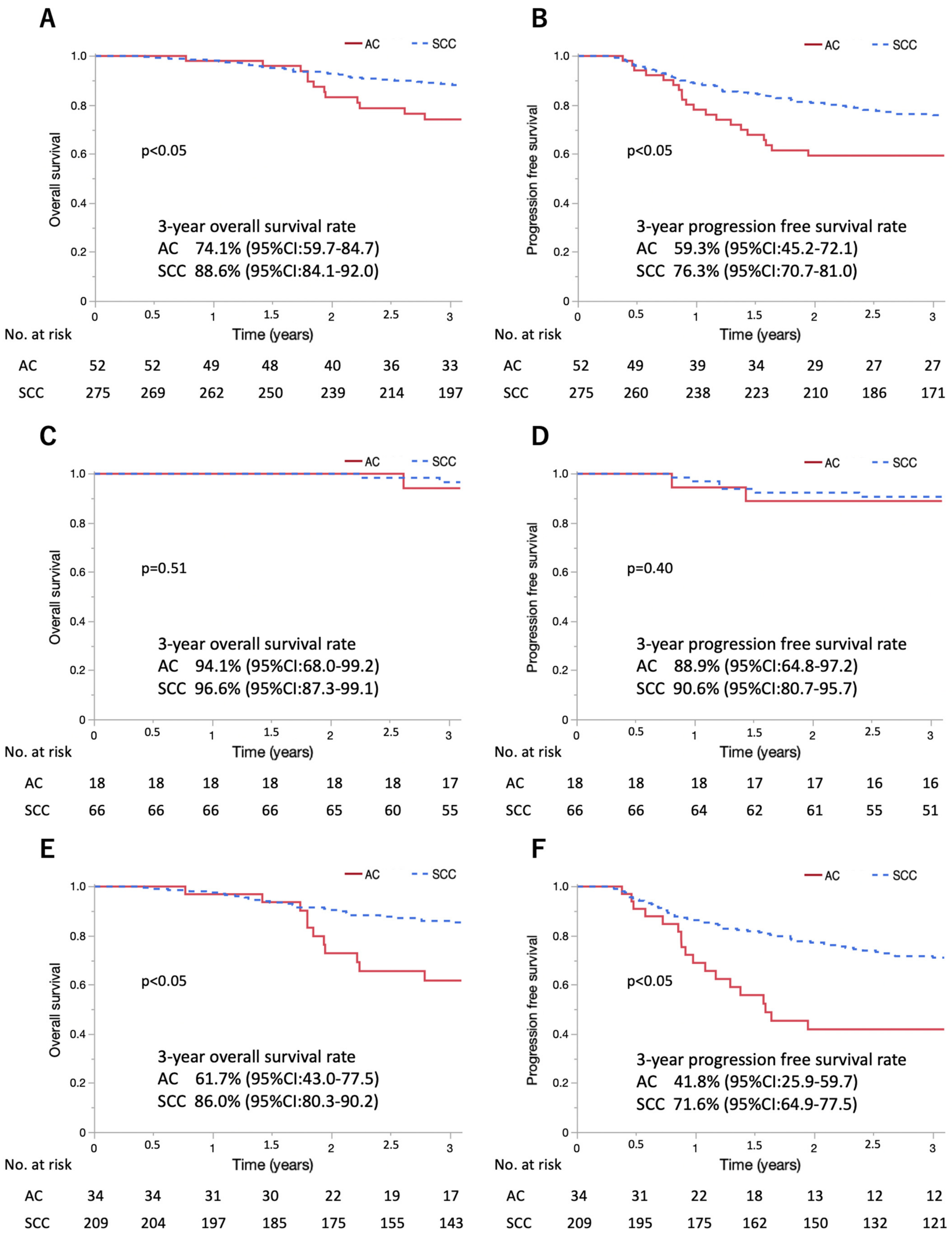

Supplementary Figure S1. The Kaplan–Meier curves of overall survival (OS) (A) and progression-free survival (PFS) (B) stratified by adenocarcinoma (AC) and squamous cell carcinoma (SCC) of the International Federation of Gynecology and Obstetrics (FIGO) stage. The Kaplan–Meier curves of OS (C) and PFS (D) stratified by AC and SCC of FIGO stage IA–IIA. The Kaplan–Meier curves of OS (E) and PFS (F) stratified by AC and SCC of FIGO stage IIB–IVA.

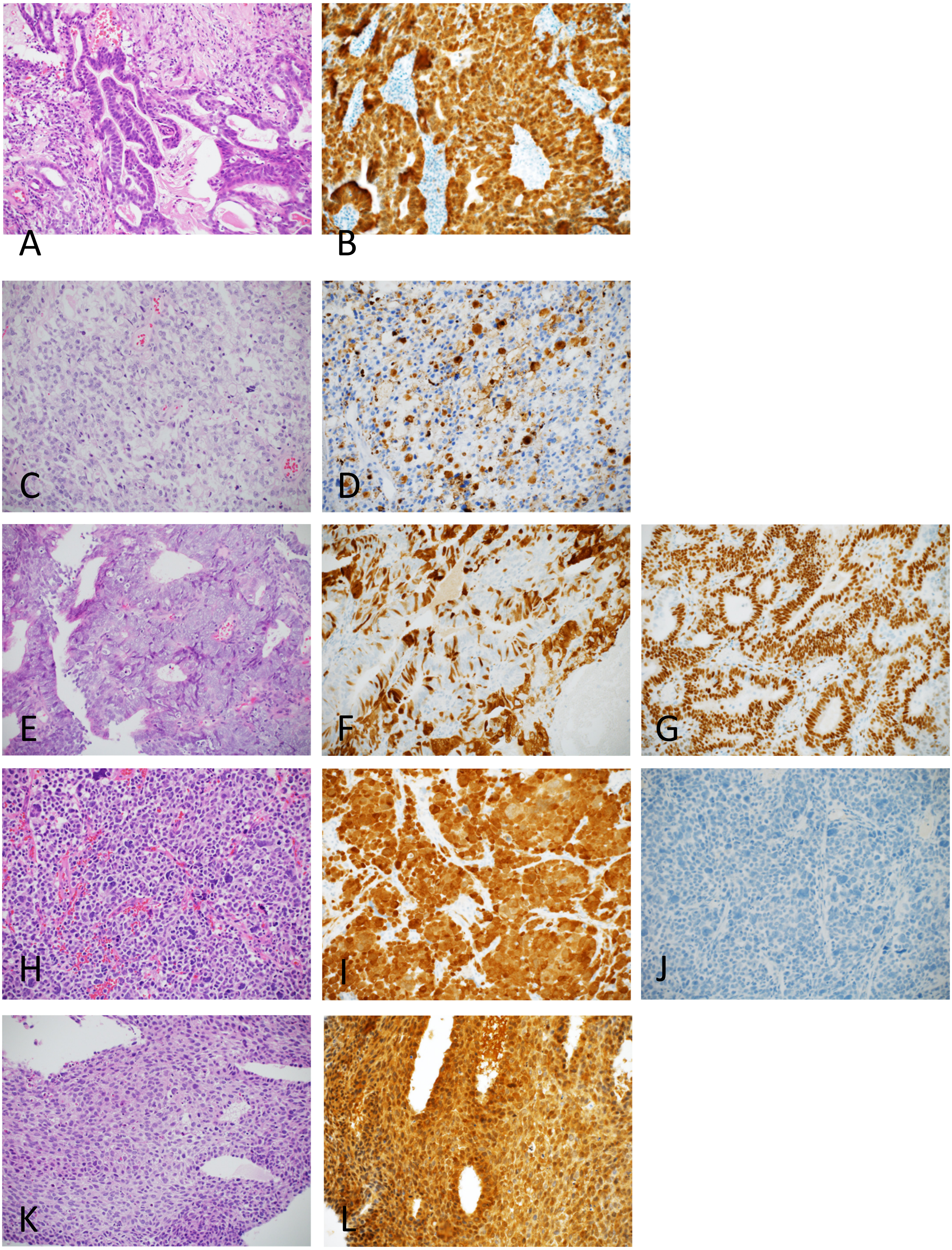
A
B
C
D
E
F
G
H
I
J
K
L

Supplementary Figure S2. Pathological images of several types of cervical adenocarcinoma.

Adenocarcinoma, usual type was stained with hematoxylin and eosin (H&E) (A) and p16 (B). Adenocarcinoma, clear cell type was stained with H&E (C) and Napsin A (D). Endometrioid adenocarcinoma was stained with H&E (E), p16 (F), and estrogen receptor (G). Poorly differentiated adenocarcinoma was stained with H&E (H), p16 (I), and p40 (J). Adenosquamous cell carcinoma was stained with H&E (K) and p16 (L).

.

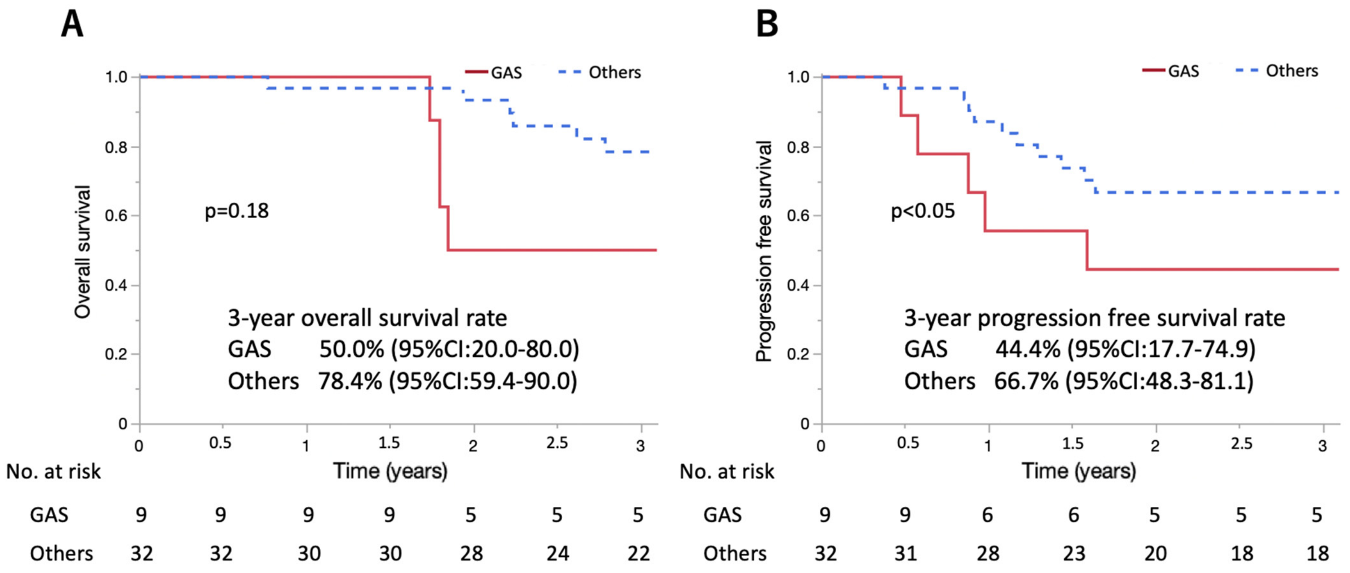

Supplementary Figure S3. (A) The Kaplan–Meier curves of overall survival stratified by gastric-type adenocarcinoma (GAS) and others. (B) The Kaplan–Meier curves of progression-free survival stratified by GAS and others.
